# Comparative Evaluation of Postbiotic Preparation Methods for Antibacterial Activity in Fresh Cheese Applications

**DOI:** 10.3390/foods15010006

**Published:** 2025-12-19

**Authors:** Joanna Gajewska, Arkadiusz Zakrzewski, Zuzanna Byczkowska-Rostkowska, Sylwester Czaplicki

**Affiliations:** 1 Department of Food Microbiology, Meat Technology and Chemistry, Faculty of Food Science, University of Warmia and Mazury in Olsztyn, Plac Cieszyński 1, 10-726 Olsztyn, Poland; 2 Chair of Food Plant Chemistry and Processing, Faculty of Food Science, University of Warmia and Mazury in Olsztyn, Plac Cieszyński 1, 10-726 Olsztyn, Poland

**Keywords:** postbiotics, lactic acid bacteria, biopreservation, fresh cheese, antimicrobial activity

## Abstract

Dairy products from unpasteurized milk produced using traditional production methods may contain many groups of microorganisms, including *Staphylococcus aureus* and *Listeria monocytogenes*. Therefore, the use of postbiotics as an alternative preservation method may be important for improving the safety of these products. Therefore, the main aim of the research conducted was to isolate and identify lactic acid bacteria and prepare postbiotics from selected strains using four different methods, sterilization, pasteurization, sonication and pascalization, to determine their antibacterial properties. The antilisterial and antistaphylococcal activities of the prepared postbiotics were investigated in vitro and in a fresh cheese model. The obtained results showed that the most effective method of postbiotic preparation was pascalization. Both, the results of the MRS medium and the challenge test confirmed that postbiotics produced through pascalization exhibited antistaphylococcal activity. This study may help identify more effective biopreservation compounds to combat pathogens in food products, particularly in dairy products.

## 1. Introduction

Nowadays, consumers are increasingly paying attention to products that are minimally processed and contain a lower quantity of additives and chemical substances, which are called “clean label” products [1]. For this reason, in recent years, there has been an increase in the consumption of raw milk and raw milk products sourced from regional markets and local farms [2].

Cheeses made through traditional methods from unpasteurized milk are characterized by a heterogeneous microbiota, which, on the one hand, determines the positive characteristics of the product, such as taste and aroma; however, on the other hand, it can be a source of bacteria that are dangerous to human health. Foodstuffs, including artisanal cheeses from unpasteurized milk, are a good habitat for pathogenic microorganisms such as *Staphylococcus aureus* and *Listeria monocytogenes* [3].

*S. aureus* is a common animal pathogen found in the dairy industry. It is responsible for food poisoning caused by staphylococcal enterotoxins. The consumption of enterotoxins with food may cause nausea, vomiting, and abdominal cramps [4]. In addition, it produces enzymes such as hemolysins and leukocidins, which increase the potential virulence of *S. aureus*. These strains are capable of producing biofilms, a process determined by various genetic factors, which enables the survival of bacterial cells in the dairy industry [2].

*L. monocytogenes* causes listeriosis, which is the one of the most dangerous diseases for pregnant women, newborns, people with immune deficiencies, and the elderly [5]. In 2021, the disease had the highest death and hospitalization rates among zoonoses under surveillance in the EU. *L. monocytogenes* is an environmental microorganism that, when transmitted through food, represents one of the most significant threats to the food industry [6]. This microorganism is capable of thriving and proliferating in low-temperature and high-salt environments [7]. These parameters occur during the production and storage of artisanal cheeses.

The change in consumers’ approaches to food processing, particularly regarding the use of preservatives, has created a need for alternative methods of contamination and spoilage prevention [8]. Chemical preservatives are generally unsuitable for artisanal cheeses, as their use would compromise the traditional character of products made solely from natural, wholesome ingredients, and their excessive intake has been linked to gut microbiota disruption and health risks. While pasteurization provides important benefits, such as reducing the microbial load and prolonging milk shelf-life, it also induces heat-related changes in milk components. In particular, whey proteins undergo denaturation and form complex interactions with casein micelles, minerals, and fat globules. These transformations alter both the biochemical and microbiological processes involved in milk acidification and cheese ripening. As a result, the unique flavor, aroma, and texture typical of raw milk cheeses cannot be fully reproduced when pasteurized milk is used [9]. Due to specific and survival-challenging conditions, the delivery of living microorganisms, probiotics, throughout processing and storage is very difficult. Postbiotics can be non-viable whole cells, their fragments, metabolites, or a combination of these, and can exhibit a broad antimicrobial activity and have the capacity to inhibit many pathogens [10]. There are many methods for postbiotic preparation that must ensure the loss of cell viability. The most frequently researched methods include sterilization, pasteurization, sonication, high pressure, and heat. The chosen technique can significantly impact the properties of the final product [11]. In recent years, postbiotics have attracted a growing interest not only in biomedical research but also in the food industry, where they are increasingly considered for use in natural preservation strategies. Regulatory authorities such as the EFSA and FDA have begun acknowledging postbiotics as promising safe bioactive compounds, although clear regulatory frameworks for their use in foods are still emerging [12]. It should be emphasized that, although the antimicrobial properties of postbiotics have been widely studied in vitro, their application in real food matrices, particularly in fresh or artisanal cheeses, is still limited. Postbiotics represent a promising natural alternative to chemical preservatives, capable of inhibiting pathogens while maintaining product quality. However, comparisons of different postbiotic preparation methods evaluating antimicrobial efficacy are limited. This study, therefore, addresses this gap by assessing multiple postbiotic preparation methods and their potential to enhance the microbiological safety of fresh cheeses, providing insights for practical applications in the dairy industry.

The aim of this study was to compare various methods to identify the most effective approach to postbiotic preparations from lactic acid bacteria (LAB), as well as to assess their potential application in reducing pathogenic microorganisms in a fresh cheese model.

## 2. Materials and Methods

### 2.1. Strains

#### 2.1.1. Lactic Acid Bacteria Isolation and Identification

The lactic acid bacteria used in this study were isolated from artisanal cheeses made from unpasteurized milk (*n* = 11), collected from a local market in the northeastern part of Poland, and commercially available probiotic supplements available on the Polish market (*n* = 3) (manufacturers: Sanprobi Sp. z o.o. Sp.k., Szczecin, Poland; Chiesi Poland Sp. z o.o., Warsaw, Poland). All probiotic supplements contained a maximum of two LAB strains. Briefly, 10 g of each cheese or one tablet of probiotic supplement was mixed with 90 mL of sterile 0.9% (*w*/*v*) saline and homogenized for 60 s using a stomacher (Masticator Homogenizator, IUL S.A., Barcelona, Spain). Then, a series of ten-fold dilutions were prepared and streaked onto de Man, Rogosa, and Sharpe agar (MRS) (Merck, Darmstadt, Germany) for lactobacilli and M17 agar (Merck, Darmstadt, Germany) for lactococci and enterococci. Samples were incubated under anaerobic conditions at 30 °C for 24 h. After incubation, typical colonies (small- to medium-sized, circular, convex, and white to creamy colonies) were picked and cultured on MRS or M17 agar. When pure strains were obtained, they were passaged onto the base medium and stored at −80 °C in the Microbank system (PRO-LAB diagnostic, Richmond Hill, ON, Canada) until further analysis. Strains were identified using VITEK^®^MS (bioMérieux, Marcy l’Etoile, Lyon, France) according to the manufacturer’s protocol, as described previously [13]. As MALDI-TOF MS analysis does not distinguish between *L. pentosus* and *L. plantarum*, differentiation was performed using the RT-PCR method, with primers and reaction conditions described previously [14] (Table S1).

#### 2.1.2. Pathogens Strains

The study included a total of four pathogenic strains, with two strains each of *S. aureus* and *L. monocytogenes*, isolated from artisanal cheeses belonging to the Department of Food Microbiology, Meat Technology and Chemistry collection. All isolates were stored in Microbank (PRO-LAB diagnostic, Richmond Hill, ON, Canada) at −80 °C. Before analysis, strains were resuscitated in Tryptic Soy Broth (TSB) (Merck, Darmstadt, Germany) and incubated at 37 ± 1 °C for 24 h.

### 2.2. Antibiotic Susceptibility Testing

The antibiotic resistance of isolated strains was determined using the Kirby–Bauer disk diffusion method. The following antibiotics, as proposed by EFSA guidance, were used for analysis: ampicillin (AMP-10 μg), vancomycin (VA-30 μg), gentamicin (CN-10 μg), kanamycin (K-30 μg), streptomycin (S-10 μg), erythromycin (E-15 μg), clindamycin (DA-10 μg), tetracycline (TE-30 μg), and chloramphenicol (C-30 μg) (Oxoid, Basingstoke, UK) [15]. Suspensions were prepared from fresh 24 h cultures to a 0.5 McFarland standard in sterile 0.9% saline. The suspensions were then streaked onto Mueller–Hinton agar (Merck, Darmstadt, Germany) using sterile swabs, and the antibiotic disks were transferred to agar using a disk dispenser. The plates were incubated for 24 h at 30 ± 1 °C. After incubation, zones of bacterial growth inhibition were measured and interpreted according to the breakpoints proposed in CLSI guidelines. Additionally, for strains with sequenced genomes, the minimum inhibitory concentrations (MICs) for lincomycin and fosfomycin were determined through a microbroth dilution according to ISO 20776-1 [16], using 96-well flat-bottom polystyrene plates, in cases where resistance genes were present.

### 2.3. Antibacterial Activity of Lactic Acid Bacteria

The antibacterial activity of lactic acid bacteria was determined for two strains of *L. monocytogenes* and two strains of *S. aureus.* The antimicrobial activity of lactic acid bacteria was determined after neutralization to pH = 7 with 20% (*v*/*v*) NaOH using the agar well diffusion assay. First, a bacterial suspension (0.5 McFarland) of each pathogen was prepared and then streaked using a sterile cotton swab over nutrient agar (Merck, Darmstadt, Germany) adjusted to 2% agar plates. Next, 100 µL of each sample of LAB was placed in 6 mm wells on prepared plates. The plates were then incubated at 37 ± 1 °C for 24 h, and the diameter of the growth inhibition zone was measured with a ruler to the nearest millimeter. Strains were classified according to their antimicrobial activity based on the diameter of the inhibition zone, no activity (≤11 mm), mild (11–16 mm), strong (17–22 mm), and strong activity (>23 mm), as described by [17]. Strains with the strongest antibacterial properties were selected for further analysis.

### 2.4. Preparation of Postbiotics

LAB strains were cultured in 500 mL MRS broth and incubated at 30 ± 1 °C for 24 h. The culture was divided into five equal subsamples of 100 mL. To obtain postbiotics, the following inactivation treatments were applied according to the literature data [18]: (1) sterilization for 15 min at 121 °C; (2) pasteurization for 30 min at 80 °C, which causes cell membrane damage, loss of intracellular nutrients and ions, ribosome aggregation, DNA filament rupture, and protein coagulation; and nonthermal treatments: (3) high pressure—the analyses were performed using the high-pressure single-chamber U4040 instrument (IWC PAN, Warsaw, Poland, Unipress Equipment Division); the used pressures were 500 MPa and 600 MPa for 10 min, which results in cell membrane damage, protein denaturation, and a decrease in intracellular pH [18]; (4) sonication—the analysis was performed using an ultrasonic Homogenizer UP200St (Hielscher, Germany) with the following parameters (20 kHz; 180 W; 5 min), based on the literature data [18], causing cell wall rupture, membrane disruption, DNA damage, and free radical generation. After treatments, the bacterial suspension was centrifuged for 10 min at 6000 rpm. The harvested cell pellets of isolates were purified twice and suspended in PBS buffer. Both the supernatant (cell-free fraction) and pellet (treated bacterial cells) of each strain were used for further analysis.

### 2.5. Chemical Composition of Postbiotics

A volume of 5 mL of the sample (cell-free supernatant) was placed into a 20 mL headspace screw top vial, and closed vials were incubated at 40 °C for 5 min and shaken at 500 rpm. After incubation, the silicone septum was pierced with a syringe, and 2.5 mL of the headspace was injected into the gas chromatographic system. Volatile compounds were analyzed using an 8890 Gas Chromatograph system (Agilent Technologies, Santa Clara, CA, USA) in combination with a MPS autosampler (Gerstel, Mülheim an der Ruhr, Germany) and an Agilent 7000D QQQ mass detector (Agilent Technologies, Santa Clara, CA, USA). The compounds were separated in a DB-624 (J&W Scientific, (Agilent Technologies, Santa Clara, CA, USA), 30 m, 0.25 mm i.d., 1.4 μm film thickness), working with the following temperature program: 40 °C; hold for 5 min; 11 °C /min up to 80 °C; 22 °C /min up to 250 °C; hold for 2 min. The temperatures for the injection port, ion source, quadrupole, and interface were set at 200, 230, 150, and 230 °C, respectively. Mass spectra were obtained through the electron impact at 70 eV in the scan range of *m*/*z* 10–200. Detected compounds were identified by comparing their mass spectra with the NIST MS library v.2017 (Scientific Instrument Services, Palmer, MA, USA).

### 2.6. Antibacterial Activity of the Prepared Postbiotics

To initially screen the large number of postbiotic samples, the agar well diffusion method was used as a preliminary step to narrow down the selection for further testing. Briefly, pathogen-inoculated agar plates were prepared by spreading a 0.5 McFarland standard bacterial suspension over the surface. Wells (6 mm in diameter) were punched into the agar, and 100 µL of each postbiotic sample was added to each well. Plates were incubated at 37 ± 1 °C for 24 h, and the diameter of the inhibition zone was measured to assess antimicrobial activity. Based on these results (Table S2), only the most active postbiotics were evaluated using the broth microdilution method to determine their MIC values. The minimum inhibitory concentrations (MICs) of the prepared postbiotics against *S. aureus* (*n* = 2) and *L. monocytogenes* (*n* = 2) were determined using a microtiter plate assay, according to the CLSI protocol [19]. Firstly, bacterial suspensions of the tested strains in 0.9% (*v*/*v*) physiological fluid were adjusted to 0.5 McFarland. Next, two-fold dilutions of postbiotics (50 µL) and prepared pathogen suspensions (50 µL) were added to each well in a V-bottom microtiter plate (Promed, Torreglia, Italy). The assay was performed in triplicate. Wells containing bacterial suspensions were used as positive controls, while sterile broth was used as a negative control. The plates were incubated at 37 ± 1 °C for 24 h. MIC was determined as the lowest concentration that completely inhibited visible bacterial growth. The MIC results obtained were the average of the readings from the replicates.

### 2.7. Analysis in Fresh Cheese Model

**Figure 1:**
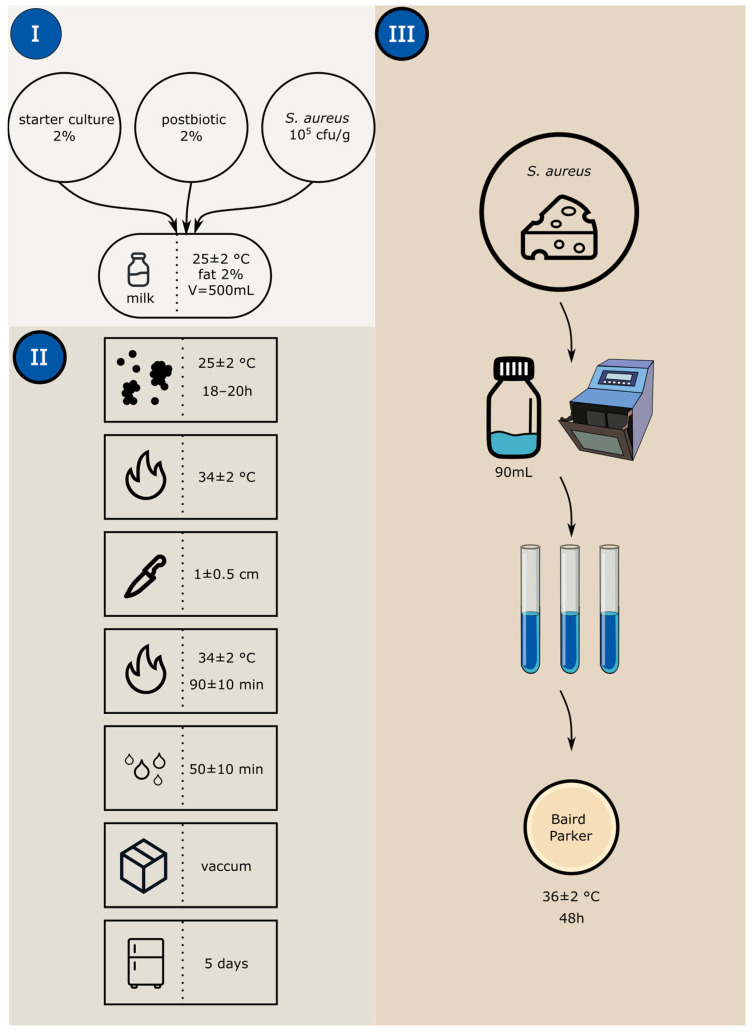
Schematic of cheese making. (I) Preparation of test and control samples, (II) Cheese production procedure, (III) Sampling for pathogen count control.

Owing to the characteristics of the selected cheese model, *S. aureus* strains were used in the analyses. *L. monocytogenes* was not included at this stage, as the physicochemical conditions of the tested cheese model are unfavorable for its growth. The choice of an artisanal cheese model was further supported by a recently published meta-analysis, which showed that as many as 42.81% of artisanal cheese samples were contaminated with *S. aureus* [20], underscoring the relevance of this product for studying this pathogen. In addition, our aim was to evaluate the efficacy of postbiotics incorporated directly into the raw material, i.e., as a preventive measure integrated into the production process, rather than as agents intended to control post-processing cross-contamination events. The challenge test was performed according to the presented scheme (Figure 1). Raw, standardized milk was inoculated with a typical starter culture for cheese production (*Lactococcus lactis* subsp. *lactis*, *Lactococcus lactis* subsp. *cremoris*, *Lactococcus lactis* subsp. *lactis biovar. diacetylactis*, *Streptococcus thermophilus*), a selected postbiotic (2%) (*v*/*v*), calculated relative to the total volume of milk, and a co-culture with *S. aureus.* A cocktail of *S. aureus* was prepared by combining equal portions of each cultured strain (~10^9^ CFU/mL) and diluted to approximately 10^5^ CFU/mL (I). The control sample did not contain any postbiotics. The standard cheese production procedure was then followed (II), starting with coagulation at 25 ± 2 °C for 18–20; then, the coagulated milk was heated to a temperature of 34 ± 2 °C. The formed curd was cut into cubes (1 cm) and heated again to 34 ± 2 °C for 90 ± 10 min. The next step was to drain the whey for 50 ± 10 min. Then the finished cheese was vacuum-packed and stored at 4 °C for 5 days. Samples to control the number of pathogens were collected three times (III): at time 0, after adding microorganisms to the milk; immediately after cheese production; and after the end of the storage period. To determine the number, 10 g/mL of the product was taken and homogenized with 90 mL of saline, and then a series of 10-fold dilutions were made and plated onto Petri plates containing Baird-Parker agar (Merck, Darmstadt, Germany) to determine the number of *S. aureus*. The plates were incubated at 37 ± 1 °C for 48 h.

### 2.8. Color Measurements

As the postbiotics were obtained from MRS-based cultures, color evaluation was included to determine whether medium components could influence the visual characteristics of the product. Color measurements were performed using a Konica Chromameter CR-400 (Sensing Inc., Osaka, Japan), using a CRA33f tip (Konica Minolta Sensing, Inc., Osaka, Japan) with a Ø22 mm measuring hole (D65 illuminant, 10° observer). Before analysis, the device was calibrated using a white plate (No.2293304, Osaka, Japan). The color parameters of L* (lightness), a* (green–red value), and b* (blue–yellow value) of samples were determined. Hue (H) and chroma (C) were calculated using Equations (1) and (2) [21]:

(1) $$H=270{}^{\circ}+\;{tan}^{-1}\frac{b*}{a*}$$

(2) $$C=\sqrt{a^{*2}+b^{*2}}$$

### 2.9. Water Activity

The analysis was performed using the LabMaster neo (Novasina AG, Lachen, Switzerland), at 25 °C in the chamber and on slow mode, on produced cheese (control) not inoculated with *S. aureus* and *L. monocytogenes.*

### 2.10. Whole-Genomic Sequencing and Bioinformatic Analysis of Selected Isolates

Sequencing of selected isolates was carried out by the third-party company Genomed (Warsaw, Poland) as described previously [22,23]. DNA libraries were prepared using the NEBNext^®^ Ultra ™ II DNA Library Prep Kit for Illumina^®^ (New England Biolabs, Ipswich, MA, USA). The sequencing process was conducted using the MiSeq Illumina^®^ platform, employing MiSeq Reagent Kit v3 reagents (600 cycles) (MS-102-3003) and utilizing PE 2 × 300 cycles.

#### Identification of Antibiotic Resistance, Virulence Genes, and Plasmids

Three distinct databases were used to identify established antimicrobial resistance genes: the web-server ResFinder v4.1.1, developed by the Centre for Genomic Epidemiology [24,25,26], the Comprehensive Antibiotic Resistance Database (CARD) v3.2.6 [27], and the database fIDBAC server [28]. For the analysis, only hits with an identity score greater than 70% were included. The evaluation of the existence of genes encoding recognized virulence factors was conducted using VirulenceFinder v2.0.3 [26,29] and the fIDBAC server [28]. The assessment was performed with the utilization of cut-off values of 85% identity and 60% coverage length.

### 2.11. Statistical Analysis

Statistical analyses were performed using Prism 10.6.1. (Prism, GraphPad Software, San Diego). The following tests were used: one-way ANOVA, ANOVA/Friedman, and ANOVA/Wilcoxon. In all analyses, the significance level α = 0.05 was assumed.

## 3. Results

### 3.1. Strain Identification

As a result of the analyses performed, after removing duplicate strains, i.e., strains of a given species from cultures of the same source, a total of 25 strains was collected and used in further analysis. The collection contained 20 strains isolated from artisanal cheeses, 3 from probiotic supplements, and 2 references strains (*Lactobacillus acidophilus* ATCC 4356 and *Limosilactobacillus reuteri* (Table 1)). The isolated strains from artisanal cheeses belonged to eight various species from six genera: *Lactiplantibacillus*, *Lacticaseibacillus*, *Latilactobacillus*, *Leuconostoc*, *Levilactobacillus*, and *Weisella*. The most frequent isolates were *Latilactobacillus curvantus* (*n* = 5) and *Lacticaseibacillus* paracasei (*n* = 4).

### 3.2. Antimicrobial Susceptibility

**Figure 2:**
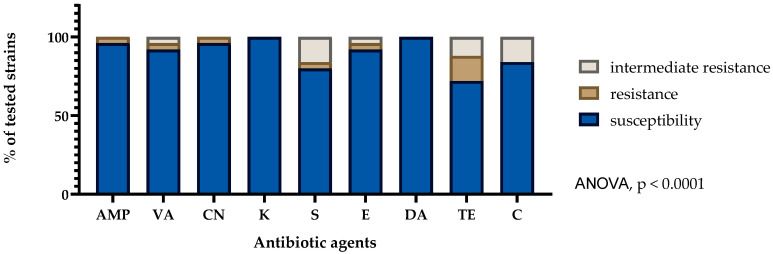
Antibiotic susceptibility patterns (%) displayed by tested lactic acid bacteria strains against different antibiotics: AMP—ampicillin; VA—vancomycin, CN—gentamycin, K—kanamycin, S—streptomycin, E—erythromycin, DA—clindamycin, TE—tetracycline, C—chloramphenicol. The distribution of susceptibility categories (susceptible, intermediate, resistant) differed significantly between antibiotics (ANOVA, *p* < 0.0001).

As a result of the conducted research, it was observed that all the tested LAB strains showed a sensitivity to kanamycin and clindamycin. The sensitivity to the remaining antibiotics ranged from 72% to 96%, with the following values: ampicillin 96%, gentamicin 96%, vancomycin 92%, erythromycin 92%, chloramphenicol 84%, streptomycin 80%, and tetracycline 72%. All strains isolated from probiotic supplements were susceptible to all tested antibiotics. Nevertheless, among the strains isolated from artisanal cheeses, four isolates (16%) were resistant to tetracycline. In addition, single isolates showed resistance to ampicillin (*L. plantarum* KN8), vancomycin (*L. paracasei* KN19), and gentamicin *(L. lactis* KN16) (Figure 2). The highest resistance was observed in two strains: KN19 (*L. paracasei*), resistant to vancomycin, erythromycin, and tetracycline, and KN8 (*L. plantarum*), resistant to ampicillin and streptomycin.

### 3.3. Antibacterial Activities of Lactic Acid Bacteria

The in vitro analysis of antibacterial properties against *S. aureus* and *L. monocytogenes* showed that isolates from artisanal cheeses were more likely to exhibit antibacterial properties against *S. aureus* (57.7%) than *L. monocytogenes* (32.7%). However, significantly larger inhibition zones were observed for *L. monocytogenes* compared to *S. aureus* (*p* < 0.05) (Table 2). The largest zones of inhibition were observed for the antibacterial properties of strains belonging to *L*. *plantarum* and *L. rhamnosus*. For *L. plantarum*, the average inhibition zone of *L. monocytogenes* was 27.3 mm, while for *S. aureus* it was 19.7 mm and for *L. rhamnosus* it was 30 and 21 mm, respectively. Five isolates—KN9, KN18, KN23, KN24, and—demonstrated the highest total inhibition zones (ranging from 85 to 102 mm) and showed activity against both *S. aureus* and *L. monocytogenes*. These strains were therefore selected for further analysis, as they exhibited broad-spectrum and strong antibacterial properties.

### 3.4. Antibacterial Activity of Tested Postbiotic

Due to the large number of postbiotics, the well diffusion method was used to reduce the number of samples for subsequent stages. In the analysis of the antibacterial properties of postbiotics, it was observed that the prepared postbiotics have the ability to inhibit the growth of only strains belonging to *S. aureus*. Additionally, of the four different methods of producing postbiotics, only those produced by the pascalization process exhibited inhibitory properties, while those prepared through sonication, pasteurization, and sterilization showed no activity (inhibition zone = 0 mm). The average growth inhibition zone for pascalization-derived postbiotics with antibacterial properties was 48 mm (*p* < 0.0001 vs. all other methods). The highest values were observed for postbiotics prepared from KN9 and KN25 strains using pascalization at a pressure of 600 MPa. The results are shown in Table S2.

The analysis also showed that bacterial pellets alone containing damaged or dead cells did not exhibit any antibacterial activity. The statistical analysis indicated that pascalization pressure (500 vs. 600 MPa) did not significantly affect the MIC values (*p* > 0.05), although a trend toward slightly lower MICs was observed for 600 MPa in some strains. The average minimum inhibitory concentration (MIC) of the postbiotics tested against *S. aureus* strains ranged from 0.39 to 50; the lowest MIC values ranged from 0.585 to 6.25% for postbiotic 9d (*L. plantarum*/pascalization 500 MPa) and postbiotic 23e (*L. plantarum*/pascalization 600 MPa). Nevertheless, the MIC values of the postbiotics prepared from the KN9 and KN24 strains (600 MPa) were comparable (0.39–9.375%). The antistaphylococcal effect at low MIC values (MIC 1.17–3.125%) was also demonstrated by postbiotic 18d with *L. paracasei* after pascalization at 500 MPa. Postbiotics prepared from *L. acidophilus* through pascalization proved ineffective, and their MIC values were >50% for each *S. aureus* strain. The analysis of variance also confirmed that antibacterial activity was strain-dependent (*p* < 0.01), with postbiotics from KN9, KN23, and KN24 exhibiting significantly lower MICs than postbiotics from KN18 and KN25. In addition, regardless of the LAB strain, all prepared postbiotics proved ineffective against *L. monocytogenes* (MIC > 50%) (Table 3).

### 3.5. Chemical Composition of Postbiotics

The chemical profiles, retention time, and the main constitutive percentages of postbiotics from different LAB strains are shown in Table 4. The GC-MS analysis revealed the presence of more than 10 compounds—including alcohols, terpenes, norisoprenoids, acids, ketones, and esters—in the postbiotics, with their proportions varying depending on the strain and the postbiotic production method. Among important volatile compounds with antimicrobial activity, the analysis demonstrated the presence of acetoin, diacetyl 2-butanone, and ethanol.

Among the tested strains, *L. rhamnosus* GG exhibited the richest and most diverse volatile profile, particularly after pascalization at 500 MPa/10 min. This strain was characterized by exceptionally high levels of acetone (14.2%) and isovaleric aldehyde (13.5%), accompanied by notable concentrations of n-hexylmethylamine (3.93%) and methylethylacetaldehyde (3.21%). The *L. plantarum* and *L. plantarum* 299v displayed a less diverse pattern, dominated by acetone (5.0–7.1%) and isovaleric aldehyde (2.2–3.5%), followed by lower amounts of methylethylacetaldehyde and 2-butanone. In *L. paracasei*, acetone (4.84–6.91%) and isovaleric aldehyde (3.53–6.34%) were also dominant, while moderate amounts of methylethylacetaldehyde (1.68–2.29%) and 2-butanone (2.10–2.44%) pointed to a similar, though less intense, metabolic activity. In contrast, *L. acidophilus* produced the lowest total concentration of volatile compounds.

### 3.6. Analysis of Cheese Model

**Figure 3:**
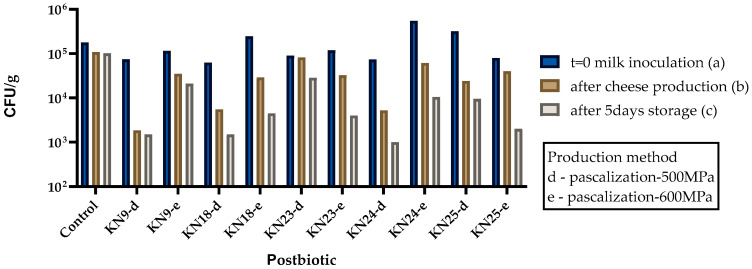
Results of *S. aureus* reduction during storage of cheese with the addition of postbiotics (cell-free supernatant). Different letters (a–c) indicate statistically significant differences between sampling times (t = 0 vs. after cheese production: *p* = 0.0326; t = 0 vs. after 5 days of storage: *p* = 0.0188; after cheese production vs. after 5 days of storage: *p* = 0.0108). d—pascalization at 500 MPa; e—pascalization at 600 MPa.

The challenge test confirmed that postbiotics produced through pascalization reduced the number of *S. aureus* in cheese. The average reduction for postbiotics usage was log 0.597 cfu/g. The greatest reduction was observed for postbiotic 25e (*L. rhamnosus*/pascalization 600 MPa), which was log 1.27 cfu/g. For the remaining strains, a reduction was observed; however, the results were <log 1 cfu/g. The reduction ranged from log 0.19 cfu/g for postbiotic 9e (*L. plantarum*/pascalization 500 MPa) to log 0.88 cfu/g for 23e (*L*. *plantarum*/pascalization 600 MPa) (Figure 3). Statistical analysis confirmed that all tested postbiotics produced through pascalization significantly reduced *S. aureus* counts compared to the control (*p* < 0.05), with variability depending on the strain and treatment intensity.

The cheeses were characterized by high lightness values (L* 87.05–88.77), corresponding to a white color. The a* values were negative (−2.79 to −2.11), indicating a slight predominance of green tones, while the b* values (7.94–9.30) reflected yellow components in the samples. The chroma (C) ranged from 8.39 to 9.62 and the hue angle (H) from 102.76° to 109.37°. The addition of postbiotics did not cause significant changes in any of the color parameters compared to the control (Table S3). The water activity of the control cheese ranged from 0.9796 to 0.9847.

### 3.7. Genomic Characteristics

**Figure 4:**
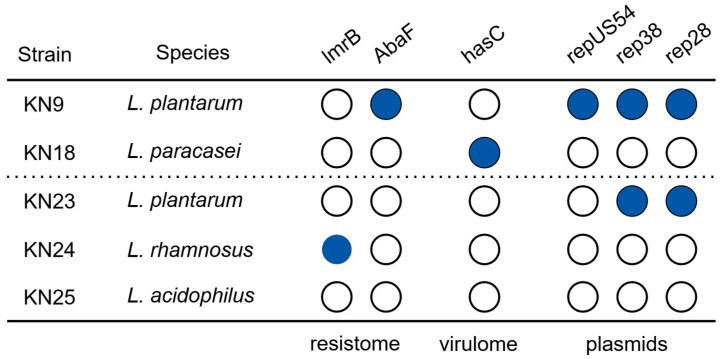
Genomic characterization of selected strains (filled shapes—presence of genes/plasmids; outlined shapes—absence of genes/plasmids).

Resistome analysis using three different databases showed that one of the isolated strains had a fosfomycin resistance gene, which was also confirmed phenotypically, as the MIC for this strain was >256 mg/mL. However, in the case of one reference strain, a lincomycin resistance gene was observed, and the MIC value was also high (64 mg/mL). Virulome analysis showed that only one strain of *L. paracasei* had the *has*C gene, which is typical of *Streptococcus pyogenes*. Plasmid analysis showed that only strains belonging to *L. plantarum* contained plasmids (Figure 4). The findings suggest that there is no risk associated with the use of these the strains in food, as resistance appears only for antibiotics not mentioned by The Panel on Additives and Products or Substances used in Animal Feed, and virulence genes appear sporadically [30].

Regarding the results obtained with BAGEL4, all tested strains were predicted as bacteriocinogenic strains. Most of the predicted peptide sequences were from *L. paracasei*, isolated from cheese. The peptide sequences were consistent with those of acidocin A and acidocin 8912, as well as sactipeptides, carnocin CP52, LSEI_2386, and LSEI_2163. For the second strain, *L. plantarum*, plantaricin E, plantaricin J, and sactipeptides were identified (Table S4). The tested reference strains are also expected to produce bacteriocins, including plantaricin E, plantaricin J, enterocin X, sactipeptide, acidocin J, enterolysin A, and helveticin J.

## 4. Discussion

Lactic acid bacteria (LAB) play an important role in cheese production as starter LAB in the acidification of curds and as non-starter LAB during ripening, especially in aroma formation [31]. In the present study, the largest group of isolated strains was classified as *Lactobacillaceae*. These are part of the fermentation process of dairy products and have the potential to act as probiotics [32]. LAB can produce antimicrobial agents such as organic acids, hydrogen peroxide, ethanol, diacetyl, acetaldehyde, acetoine, carbon dioxide, reuterin, reutericyclin, and bacteriocins, which exert strong antagonistic activity against many microorganisms, including pathogenic and spoilage microorganisms [33]. However, it is worth emphasizing that the qualitative and quantitative composition of these metabolites depends on the particular species or strain and its individual biochemical characteristics [34].

In our study, the volatile compounds identified in the postbiotics, such as diacetyl, acetoin, and dimethyl disulfide, are well-documented in the literature for their antibacterial properties, particularly against Gram-positive bacteria, through mechanisms including membrane disruption and interference with metabolic pathways. The presence and abundance of diacetyl and acetoin likely play a key role in the selective antibacterial activity against *S. aureus*, as these metabolites are known to inhibit bacterial growth at low concentrations without affecting *L. monocytogenes* [35].

From a safety perspective, the EFSA guideline recommended that LAB for human consumption should be tested for their antimicrobial resistance. In our study, some strains showed a resistance to tested antibiotics, including tetracycline, which was also observed by Duche et al. [36]. Strains showing antibiotic resistance were not selected for further analysis due to weak or no antibacterial properties. It is also important to ensure that strains are not a source of antibiotic resistance genes. The genome analysis of the selected strains showed no risk of their use as food additives. Although postbiotics are cell-free and non-viable, the antibiotic resistance of the producer strains was assessed to ensure biosafety and rule out the potential presence of residual DNA-carrying ARGs in postbiotic preparations. It is important to highlight that the direct use of live microorganisms in food has limitations because the growth and survival of bacterial cells are influenced by external factors such as food type, temperature, and pH [37]. Therefore, the use of postbiotics instead of LAB was an approach followed in this study. Adding postbiotics to dairy products is a novel technique for enhancing the safety of these products.

In our study, postbiotics from *L. plantarum*, *L. paracasei* and *L. rhamnosus* showed antistaphylococcal activity. The effect of *L. plantarum* from Polish regional cheeses against *S. aureus* was shown by Ołdak et al. (2020), highlighting the enzymatic profile of this species, especially the production of β-galactosidase by *L. plantarum* cells [38]. Jiang et al. (2022) demonstrated that the bacteriocins produced by *L. paracasei*, isolated from traditional Chinese yogurts and present in the cell-free supernatant, effectively inhibited *S. aureus* cells and their biofilm formation [39]. The weakest effect against *S. aureus* in our study was observed with *L. acidophilus*; however, the antistaphylococcal properties of this species have been confirmed in another study. Koohestani et al. (2018) showed that a suspension containing *L. acidophilus* cells and CFS inhibits the growth of *S. aureus* [40]. In the case of *L. rhamnosus*, antimicrobial activity is highlighted due to the production of bacteriocin-like substances that remain active after pH neutralization [41]. Yüksek et al. (2021) showed that a CFS from *L. rhamnosus* was effective even against MRSA [42].

Some studies have used postbiotics in food models; however, they have mainly focused on pasteurized milk. Interestingly, some authors managed to effectively eliminate *L. monocytogenes* in milk using postbiotics obtained from *L. acidophilus*, *L. casei*, and *L. salivarius* at concentrations of 45, 35, and 25 mg/mL, respectively, which corresponded to the minimum effective concentrations (MECs) reported in that study [37].

In our study, the antilisterial properties were not confirmed in vitro. Therefore, a comparison of the used postbiotics was not possible. Although several volatile compounds that have previously been implicated in the antimicrobial activity of LAB cell-free supernatants, such as diacetyl, acetoin, and dimethyl disulfide [43], were also detected in our postbiotic preparations, they were present only in low relative amounts (approximately 0.1–1.9% of the total volatile fraction depending on the strain; e.g., diacetyl 0.34–1.92%, acetoin 0.14%, and dimethyl disulfide ≤ 0.74%). This suggests that their absolute concentrations in the study were likely below the levels required for a measurable antilisterial effect, particularly at the nearly neutral pH used in our assays. In addition, the main inhibitory factors often associated with the antimicrobial activity of CFS against *L. monocytogenes*, namely lactic and acetic acids and hydrogen peroxide, were largely removed or inactivated during postbiotic preparation (neutralization, cell removal, and processing). The combination of reduced acid stress and low levels of antibacterial volatiles may therefore explain why postbiotics did not inhibit *L. monocytogenes* in our model, while still being sufficient to exert a selective antistaphylococcal effect. It is not possible to draw any strong conclusions; however, taken together, these findings might explain why the prepared postbiotics exerted only an antistaphylococcal effect, while no measurable inhibition of *L. monocytogenes* was observed under the tested conditions.

In addition, most studies reporting the strong antilisterial activity of LAB metabolites are based on classical cell-free supernatants obtained only through centrifugation and membrane filtration. In contrast, in our work, the postbiotics were produced using additional processing steps, including heat treatment, sonication, and high hydrostatic pressure, which may have altered the composition and/or stability of labile antimicrobial compounds. Such processing-related modifications could further attenuate the antilisterial potential of the preparations, while maintaining their selective activity against *S. aureus*. However, a further characterization of the molecular components responsible for these effects will be essential in future investigations.

The antistaphylococcal activity in milk showed that postbiotics produced from *L. plantarum* and *L. acidophilus* had the lowest minimum effective concentration (MEC < 20 mg/mL) compared to postbiotics produced from *L. paracasei* (MEC > 20 mg/mL). The authors did not neutralize postbiotics and suggested the antibacterial effects of lactic acid, laurostearic acid, and isopropylidene-3,3-dimethyl [44].

In our model using fresh cheese, the postbiotic produced from the *L. rhamnosus* GG showed the best effect. The antistaphylococcal abilities of this strain have been proven [45,46]. As a probiotic strain, it has also been used in Minas Frescal cheese, where it effectively reduced the number of *L. monocytogenes*, suggesting that the action of acids influences this feature [47].

Among the strains isolated from cheese, the postbiotic prepared from *L. paracasei* was the most effective in reducing staphylococci. It has as many as five genes encoding bacteriocins, yet none of them work effectively against *S. aureus* [48]. Since the postbiotics were neutralized, other components must be responsible for the action against *S. aureus*.

Another use of postbiotics to combat the development of spoilage microbiota was proposed by Sharafi et al. Their model was based on adding postbiotics made from *L. acidophilus* and *B. animalis* to the mozzarella marinade. The results were promising because a significantly reduced growth of mesophilic bacteria, psychrophiles, and fungi was observed. Interestingly, even the growth of LAB was limited compared to the control sample [49].

In our study, we did not focus on the analysis of genome stability because only fragments of damaged cells were added to the cheese. However, since the degree of cellular damage is unknown, the analysis of the virulome, resistome, and plasmid content was performed. Both genes (*imr*B and *aba*F) found in the strains are chromosomally encoded efflux pump genes; therefore, they have a low probability of being passed on to the cheese microbiota, or later to commensal or pathogenic bacteria in the intestines. Of the two selected strains, in one (*L. plantarum*), the presence of as many as three plasmids was observed; however, based on the results, we were unable to determine whether postbiotics prepared using pascalization contained transferable mobile genetic elements. It is worth noting that, although our study showed the antistaphylococcal activity of postbiotics, preparing postbiotic compounds in MRS broth will not be as safe as commercially prepared postbiotics due to possible changes in sensory quality. However, in this study, adding postbiotics (in low concentration) did not affect color changes in fresh cheeses. It is worth mentioning that the brown to brownish-yellow color of the postbiotics prepared in MRS broth may restrict postbiotic utilization, especially at higher concentrations, in many dairy products [50]. MRS is inherently suboptimal for food-grade postbiotic production, as it may induce undesirable sensory modifications. Khakpour1 et al. (2024) reported that postbiotics produced in MRS exhibited color changes that could limit their application in dairy matrices. The study further demonstrated that alternative media, such as whey or supplemented milk-based substrates, can produce postbiotics with a similar antimicrobial efficacy while maintaining a more favorable color and sensory profile. These findings indicate that, although MRS is commonly used in laboratory settings, it may not be suitable for industrial or food applications, and food-compatible alternative media should be considered for products where color and overall sensory qualities are critical [51].

Our study has some limitations that should be acknowledged. First, although genes encoding bacteriocins were identified in the genomes of the selected lactic acid bacteria strains, we did not perform a detailed phenotypic characterization of the specific bacteriocins responsible for the observed antimicrobial activity. Further studies involving protein isolation and functional assays are needed to confirm their expression and mechanism of action. Second, the antimicrobial activity was tested only in a single food model (fresh cheese), which limits the generalizability of the results. Future studies should evaluate the effectiveness of postbiotics in other food matrices with different physicochemical properties. Additionally, while promising antilisterial and antistaphylococcal activity was observed, the study was limited to in vitro tests and a short-term challenge test; thus, longer shelf-life studies and in vivo safety assessments would be necessary to validate the practical application of these postbiotics in real food systems. Taking the above considerations into account, postbiotics may serve as natural, label-friendly biopreservatives with potential applications in products. However, their scalability, regulatory compliance, and performance during long-term storage must be thoroughly evaluated in future research.

## 5. Conclusions

The emerging trend of eliminating synthetic preservatives has influenced researchers to develop alternative, natural methods of preservatives which will be able eradicate food-contaminating pathogens. Considering the different methods of postbiotic preparation evaluated in this study, pascalization was the most effective in terms of preserving the antistaphylococcal activity of the obtained postbiotics. Based on the conducted research, two strains belonging to the species *L. plantarum* and *L. paracasei* were selected as candidates for the preparation of postbiotics for use in the production of artisanal cheese. Both candidates have low pathogenic potential, low antibiotic tolerance, and a relatively stable genome and are effective in reducing staphylococci. However, before industrial implementation, additional studies are needed to evaluate process scalability, long-term stability, and the potential sensory effects of postbiotic addition under real production conditions.

## Figures and Tables

**Table 1:** List of strains used in this study.

Strain ID	Identification	Source
KN1	*Lactiplantibacillus plantarum*	Artisanal cheese from unpasteurized milk
KN2	*Levilactobacillus brevis*	
KN3	*Levilactobacillus brevis*	
KN4	*Latilactobacillus curvatus*	
KN5	*Latilactobacillus curvatus*	
KN6	*Lactiplantibacillus plantarum*	
KN7	*Leuconostoc mesenteroides* ssp. *cremoris*	
KN8	*Lactiplantibacillus plantarum*	
KN9	*Lactiplantibacillus plantarum*	
KN11	*Leuconostoc mesenteroides*	
KN12	*Leuconostoc mesenteroides*	
KN13	*Latilactobacillus curvatus*	
KN14	*Latilactobacillus curvatus*	
KN15	*Latilactobacillus curvatus*	
KN16	*Leuconostoc lactis*	
KN17	*Lacticaseibacillus paracasei*	
KN18	*Lacticaseibacillus paracasei*	
KN19	*Lacticaseibacillus paracasei*	
KN20	*Lacticaseibacillus paracasei*	
KN21	*Weisella confusa*	
KN22	*Lactobacillus helveticus Rossel*	
KN23	*Lactiplantibacillus plantarum* 299v (*f. Lactobacillus plantarum* 299v)	Probiotic supplements
KN24	*Lacticaseibacillus rhamnosus GG* (*f. Lactobacillus rhamnosus GG*)	
KN25	*Lactobacillus acidophilus ATCC 4356*	Reference strains
KN26	*Limosilactobacillus reuteri*	

**Table 2:** Zones of inhibition [mm] indicating the antibacterial activity of lactic acid bacteria isolates against *L. monocytogenes* and *S. aureus* strains.

Strain	*S. aureus*		*L. monocytogenes*		Σ (Total Inhibition)	Classification
	Sa-21	Sa-121	N26	N28		
KN1	18	17	25	25	85	Moderate activity
KN2	0	15	7	0	22	No activity
KN3	0	0	0	0	0	No activity
KN4	0	0	0	0	0	No activity
KN5	0	0	0	0	0	No activity
KN6	0	0	0	0	0	No activity
KN7	0	17	0	0	17	No activity
KN8	0	0	0	0	0	No activity
KN9	22	21	29	29	101	Strong activity
KN11	0	11	0	0	11	No activity
KN12	0	0	0	0	0	No activity
KN13	0	14	0	0	14	Mild activity
KN14	0	0	0	0	0	No activity
KN15	20	22	0	0	42	No activity
KN16	11	16	0	0	27	No activity
KN17	0	0	0	0	0	No activity
KN18	20	20	24	21	85	Moderate activity
KN19	17	15	23	22	77	Moderate activity
KN20	16	17	23	23	79	Moderate activity
KN21	15	15	0	0	30	No activity
KN22	11	11	0	0	22	No activity
KN23	20	20	27	29	96	Strong activity
KN24	20	22	30	30	102	Strong activity
KN25	22	20	21	23	86	Moderate activity
KN26	29	28	0	0	57	Mild activity

Classification: no activity (≤11 mm), mild activity (11–16 mm), moderate activity (17–22 mm), and strong activity (>23 mm) [14].

**Table 3:** Minimum inhibitory concentrations of prepared postbiotics (cell-free supernatant).

Strain		Postbotic Preparation Method	MIC [% *v*/*v*]	
			*S. aureus*	*L. monocytogenes*
KN9	*L. plantarum*	500 MPa/10 min	0.585–6.25	>50
		600 MPa/10 min	0.78–9.375	
KN18	*L. paracasei*	500 MPa/10 min	1.17–3.125	
		600 MPa/10 min	3.125–12.5	
KN23	*L. plantarum* 299v	500 MPa/10 min	1.25–50	
		600 MPa/10 min	0.585–6.25	
KN24	*L. rhamnosus GG*	500 MPa/10 min	2.343–50	
		600 MPa/10 min	0.39–6.25	
KN25	*L. acidophilus* ATCC^®^ 4356	500 MPa/10 min	>50	
		600 MPa/10 min	>50	

**Table 4:** Results of AGC-MS analysis of volatile compounds from obtained postbiotics (cell-free supernatant) from lactic acid bacteria.

Compounds (%)	*L. plantarum*		*L. paracasei*		*L. plantarum* 299v		*L. rhamnosus GG*		*L. acidophilus*	
	500 MPa	600 MPa	500 MPa	600 MPa	500 MPa	600 MPa	500 MPa	600 MPa	500 MPa	600 MPa
	%		%		%		%		%	
Acetone	6.03	7.15	6.91	4.84	5.35	5.02	14.2	7.39	5.56	7.08
Isovaleric aldehyde	2.25	2.87	6.34	3.53	3.53	2.85	13.5	8.29	3.68	4.81
n-Hexylmethylamine	1.55	1.9	1.77	4.32	0.91	1.37	3.93	2.24	0.73	1.04
2-Butanone *	1.18	1.86	2.1	2.44	1.11	1.64	1.89	2.67	0.93	1.11
Methanethiol	0.64	nd	nd	nd	nd	0.25	nd	nd	nd	nd
2-tert-Butyl-3-methyloxirane	0.64	0.51	1.73	0.67	0.55	0.32	0.78	1	0.38	nd
Diacetyl	0.56	0.79	0.73	1.48	0.73	0.7	1.92	1.1	0.64	0.34
Isopropylaldehyde	0.44	0.52	1.64	1.35	0.61	0.47	2.3	1.43	0.6	0.88
n-Butan-1-ol	0.43	0.44	0.33	0.15	0.44	0.36	nd	nd	0.38	nd
Ethanol	0.36	0.46	0.78	0.97	0.33	0.51	0.81	1.01	0.27	0.23
Methylethylacetaldehyde	0.34	0.45	2.29	1.68	0.75	0.47	3.21	2.21	0.85	1.16
Dimethyl disulfide	0.31	0.3	0.45	0.29	0.39	0.3	0.74	nd	nd	0.16
2-Heptanone	0.15	0.1	nd	nd	0.18	nd	0.22	nd	nd	nd
Acetoin	0.14	nd	nd	nd	nd	nd	nd	nd	nd	nd
1-Isobutyl-1-methyl-2-oxohydrazine	0.11	0.13	nd	nd	0.12	nd	0.17	nd	nd	nd
Benzaldehyde	nd	nd	0.28	nd	0.17	nd	0.5	0.12	nd	nd
Ethyl acetone	nd	nd	nd	nd	nd	nd	0.13	nd	nd	nd

The lowest and highest values are represented by a gradient of lightness, ranging from light (lowest) to dark (highest), respectively; nd—not detected. * Statistical significance in differences in the amount of compound obtained after pascalizations d and e (*p* < 0.05), where values were higher after pascalization at 600 MPa/10 min.

## Data Availability

Data are contained within the article and Supplementary Materials.

## Supplementary Materials

The following supporting information can be downloaded at https://www.mdpi.com/article/10.3390/foods15010006/s1. Table S1: Primers used in RT-PCR. Table S2: Inhibition zones [mm] of the growth of *L. monocytogenes* and *S. aureus* strains under the influence of produced postbiotics. Table S3: The effect of postbiotic additives on the color of cheeses. Table S4: Bacteriocin genes among selected LAB based on BAGEL analysis.

## Abbreviations

The following abbreviations are used in this manuscript:

ARG	Antibiotic Resistance Gene
BAGEL	Bacteriocin Genome Mining Tool
CFS	Cell-Free Supernatant
CLSI	Clinical and Laboratory Standards Institute
CFU	Colony Forming Unit
EFSA	European Food Safety Authority
GC-MS	Gas Chromatography–Mass Spectrometry
LAB	Lactic Acid Bacteria
MRS	De Man, Rogosa, and Sharpe
MIC	Minimum Inhibitory Concentration
MEC	Minimum Effective Concentration
TSB	Tryptic Soy Broth
PBS	Phosphate-Buffered Saline
