# Supplementary material for: Comparative Evaluation of Postbiotic Preparation Methods for Antibacterial Activity in Fresh Cheese Applications
Source: Foods. 2025 Dec 19;15(1):6. doi: 10.3390/foods15010006 (PMC12785956; doi:10.3390/foods15010006)
Supplement: Supplementary file 1 [file foods-15-00006-s001.zip › Table S1. Primers used in study. .pdf]

Table S1. Primers used in RT-PCR.

Target species	Primer name	Sequence (5'-3')	Size (bp)
L. plantarum* subsp. *plantarum	Plantarum_F	CGG CAA CAA GCC ACT AAA CT	120
	Plantarum_R	GAT AAT TAG CGG CTG CCT GA	
L. plantarum* subsp. *argentoratensis	Argentoratensis_F	TTC TTG ATG GCC CGG GTG TT	143
	Argentoratensis_R	GGC TGG ACC ATG GCT AAG AA	
L. paraplantarum	Paraplantarum_F	TTA TTC AAG CCG TCG GAG TG	128
	Paraplantarum_R	TCG CTG GTG CTA ATG CAA TG	
L. pentosus	Pentosus_F	GCG GTA TCG ATT CGA TTG GT	145
	Pentosus_R	TGA TGT CAA TCG CCT CTT GG	
