# Supplementary material for: Comparative Evaluation of Postbiotic Preparation Methods for Antibacterial Activity in Fresh Cheese Applications
Source: Foods. 2025 Dec 19;15(1):6. doi: 10.3390/foods15010006 (PMC12785956; doi:10.3390/foods15010006)
Supplement: Supplementary file 1 [file foods-15-00006-s001.zip › Table S3.pdf]

Table S3. The effect of postbiotic additives on the colour of cheeses (mean value ± standard error of the mean).

	Cheese samples with addiction of prepared postbiotics:										
**Colour Parameters**	Control	9d	9e	18d	18e	23d	23e	24d	24e	25d	25e
**L***	88.77 ± 0.30	87.05 ± 0.34	87.10 ± 0.34	88.23 ± 0.30	88.10 ± 0.23	88.26 ± 0.23	88.53 ± 0.16	88.44 ± 0.23	88.47 ± 0.15	87.90 ± 0.23	87.92 ± 0.42
**a***	-2.79 ± 0.09	-2.11 ± 0.10	-2.50 ± 0.18	-2.41 ± 0.06	-2.22 ± 0.10	-2.22 ± 0.10	-2.53 ± 0.05	-2.24 ± 0.07	-2.47 ± 0.22	-2.23 ± 0.10	-2.46 ± 0.12
**b***	7.94 ± 0.24	9.30 ± 0.47	9.18 ± 0.18	8.42 ± 0.67	8.49 ± 0.11	8.49 ± 0.11	8.63 ±0.16	8.08 ± 0.41	9.30 ± 0.28	8.71 ± 0.10	8.80 ± 0.44
**C**	8.42 ± 0.19	9.54 ± 0.48	9.53 ± 0.22	8.76 ± 0.62	9.47 ± 0.16	8.77 ± 0.11	8.99 ± 0.14	8.39 ± 0.40	9.62 ± 0.30	8.99± 0.12	9.12 ± 0.43
**H**	109.37 ± 1.1	102.76 0.07	105.24 ± 0.79	106.04 ± 1.59	105.02 ± 0.93	104.64 ± 0.6	106.33 ± 0.6	105.48 ± 0.58	104.86 ± 1.12	104 ±.36 0.48	105.61 ± 0.93
